# Improvements in Cardiopulmonary Exercise Test Results in Atrial Fibrillation Patients After Radiofrequency Ablation in Kazakhstan

**DOI:** 10.3390/diagnostics14212355

**Published:** 2024-10-22

**Authors:** Akmaral Beisenbayeva, Makhabbat Bekbossynova, Abay Bakytzhanuly, Uldana Aleushinova, Feruza Bekmetova, Assel Chinybayeva, Ayan Abdrakhmanov, Altynay Beyembetova

**Affiliations:** 1 Corporate Fund ”University Medical Center”, Heart Center, Astana 010000, Kazakhstan; 2 Department of Cardiology, Non-Profit Joint Stock Company “Medical University Astana”, Astana 010000, Kazakhstan; chena@bk.ru (A.C.); ayan-3@mail.ru (A.A.); 3 Republican Specialized Scientific Practical-Medical Center of Cardiology, Astana 010000, Kazakhstan; 4 Department of Medicine, School of Medicine, Nazarbayev University, Astana 010000, Kazakhstan; beyembetova@gmail.com

**Keywords:** atrial fibrillation, arrythmia, heart failure, radiofrequency ablation, cardiorespiratory exercise testing, CPET, VO_2_ max, METs, Nt-proBNP, 6-min walking test

## Abstract

This prospective study evaluates the impact of radiofrequency ablation (RFA) on cardiorespiratory indicators in patients with long-standing persistent atrial fibrillation admitted to the Heart Center UMC between January 2022 and April 2024 in Astana, Kazakhstan. The study aims to assess the functional cardiac benefits of RFA. Out of 717 registered atrial fibrillation patients, 104 were examined before and 3 months after ablation, focusing on cardiorespiratory parameters. A before-and-after analysis using linear mixed models was applied to evaluate changes in cardiorespiratory parameters post-RFA. Significant improvements were noted across various measures. VO_2_ max increased from 11.5 ± 4.4 mL/kg/min to 18.0 ± 4.5 mL/kg/min (*p* < 0.001). Oxygen uptake improved from 7.2 ± 2.6 mL/beat to 11.0 ± 3.4 mL/beat (*p* < 0.001). The 6-min walking test distance rose from 306 ± 82 m to 400 ± 48 m (*p* < 0.001). METs increased from 4.4 ± 1.6 to 8.0 ± 1.3 (*p* < 0.001). Heart rate at peak exercise decreased from 175.5 ± 18.6 to 147.2 ± 12.3 beats per minute (*p* < 0.001). NT-proBNP levels decreased from 1357 ± 1182 to 415 ± 339 pg/mL (*p* < 0.001). Patients with persistent atrial fibrillation undergoing RFA showed functional improvements in CPET indicators such as VO_2_ max, METs, O_2_ pulse, heart rate, and the 6-min walking test. Improvements were also seen in Nt-proBNP analysis. These results emphasize the need for longitudinal follow-up to optimize outcomes and minimize medical risks.

## 1. Introduction

Atrial fibrillation (AF) is the most prevalent and persistent cardiac arrhythmia, significantly increasing the risk of stroke, heart failure, and mortality, thereby imposing a substantial burden on healthcare systems [1,2]. Often described as a cardiovascular epidemic, AF now affects approximately 1 in 4 individuals over the age of 45, with its prevalence steadily rising worldwide [3]. In 2019, the global AF cases were estimated at 59.7 million, doubling since 1990 [3,4,5,6]. By 2050, the global burden of AF is projected to increase by 60%, with an estimated 16.08 million new cases in men and 16.85 million in women between 2030 and 2034 [5,6]. AF shares with other non-communicable diseases several risk factors, including hypertension, diabetes, obesity, and aging [6,7,8]. Additionally, AF is associated with atrial enlargement, hyperthyroidism, and genetic predispositions, which help to differentiate it from other arrhythmias [9,10]. Moreover, specific patient characteristics—such as female sex, younger age, recent onset of AF, and comorbidities like heart failure and sleep apnea—further contribute to reduced QoL [11]. Beyond the symptoms of AF, factors like perceived stress and levels of physical activity have been identified as important predictors of QoL, highlighting the need for personalized care strategies [12,13,14,15].

Many AF patients are asymptomatic, leading to underdiagnosis and delayed treatment. In Kazakhstan, official data report an AF prevalence of about 130–140 cases per 100,000 people [16]. This figure is nearly five times lower than estimates from the Global Burden of Disease study, likely due to underdiagnosis and limited access to diagnostic tools [17]. Effective management of AF necessitates a comprehensive approach, including anticoagulation, rate or rhythm control, lifestyle modifications, and multidisciplinary care. Cardiopulmonary exercise testing (CPET) is also utilized in AF management to evaluate functional capacity and mitigate exercise intolerance, ultimately enhancing cardiovascular health [18,19]. Catheter ablation has been shown to be a cost-effective strategy in AF management, particularly when compared to long-term pharmacotherapy, with potential to reduce healthcare costs, hospitalizations, and overall resource utilization [20,21,22].

While RFA is an established treatment for rhythm control in AF, there is a significant lack of data regarding its impact on cardiopulmonary function in the Central Asian population, including the Kazakh ethnic group, which constitutes the majority of Kazakhstan’s population. The predominant studies on RFA have focused on Western and other non-Central Asian populations, leaving a critical gap in our understanding of how CPET indicators changes before and after RFA affecting cardiopulmonary outcomes in this region. Given potential genetic, environmental, and healthcare access differences, it is essential to generate evidence specific to the Central Asian population to ensure more tailored and effective management of AF. For instance, the “CopenHeartRFA” RCT trial evaluated cardiopulmonary improvements and exercise capacity measured by CPET at 12 and 24 months after participation in a multidisciplinary cardiac rehabilitation program, found that patients who participated in the program had higher peak oxygen uptake (VO_2_ peak) and lower anxiety levels compared to those receiving usual care alone [23]. An Australian RCT trial reported improvements such as a reduction in peak exercise pulmonary capillary wedge pressure (PCWP), an increase in peak oxygen uptake (VO_2_), and lower N-terminal pro–B-type natriuretic peptide (NT-proBNP) levels [24]. One more Japanese study indicated cardiopulmonary functional improvements, suggesting that AF patients with reduced exercise tolerance benefit the most from ablation [25,26,27,28].

The main aim of this work is to evaluate the functional cardiac benefits of CPET by assessing its impact before and after RFA on various cardiorespiratory indicators in patients with long-standing persistent atrial fibrillation in Kazakhstan. The principal conclusion is that cardiorespiratory indicators significantly improved functional capacity and cardiac performance after RFA, as evidenced by enhanced VO_2_ max, METs, oxygen uptake, heart rate, and reduced NT-proBNP levels in patients with persistent atrial fibrillation.

## 2. Materials and Methods

### 2.1. Study Design

The prospective observational study was based on the Heart Center at the University Medical Center (UMC), Astana city, Kazakhstan, the leading heart surgery clinic in Central Asia. The study participants were recruited during the period between January 2022–April 2024.

### 2.2. Study Population

**Chart 1:**
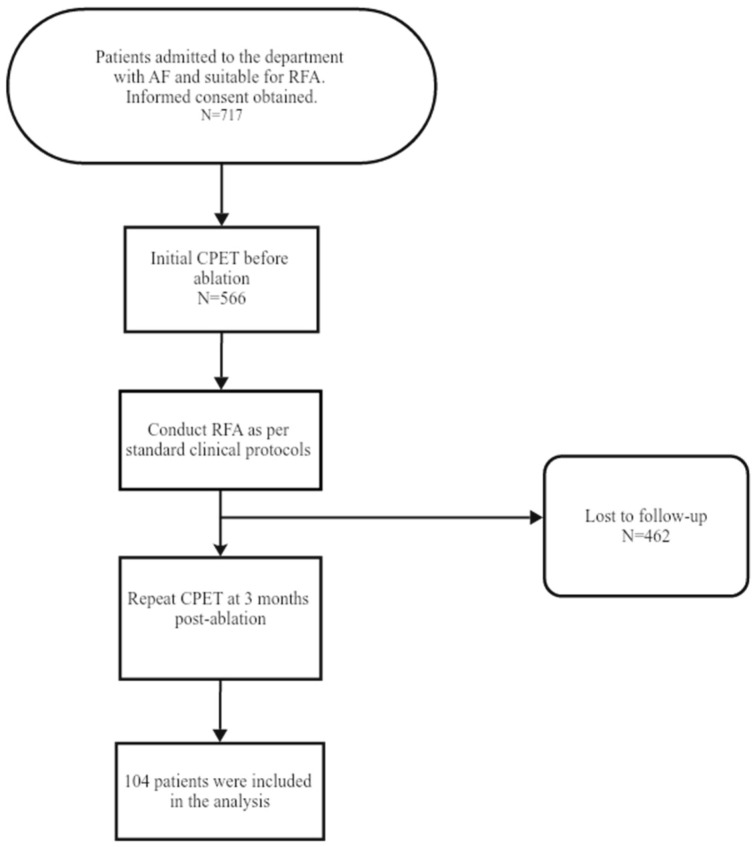
Flow-chart of selection of participants included in the analysis.

The inclusion criteria were adult patients with atrial fibrillation scheduled for radiofrequency ablation (RFA). The exclusion criteria were comorbidities (sinus rhythm, recurrent AF, left ventricular (LV) dysfunction due to coronary artery disease, idiopathic dilated cardiomyopathy, uncontrolled hypertension, previous heart surgeries); permanent pacemakers or defibrillators; less than 12 months LSPAF; sensitivity to oral amiodarone; unresistant to direct electrical cardioversion. The selection process is depicted in Chart 1.

### 2.3. CPET Preparations and Procedures

The cardiopulmonary exercise testing (CPET) measurements were collected twice before the RFA and 3 months after the procedure. The equipment used for the CPET was Schiller CS-104, Switzerland. Before the testing, patients were interviewed on particular symptoms such as painful sensations, relevant medical history, and intake of medications. Study participants were informed on the procedure and any adverse effects of the CPET and gave written consent. The patient preparation included a light meal 3 h prior to the test, comfortable and loose clothes, sports footwear, no physical exercise for 6 h prior, and avoiding strong coffee, energy drinks, and smoking tobacco products. Environmental variables, such as indoor temperature and weather conditions, were carefully calibrated to ensure consistency across all CPET sessions. However, certain patient-reported factors, such as smoking habits and adherence to prescribed medications, presented a challenge in terms of verification. Although participants were instructed to report their smoking status and medication use accurately, it is possible that some may have misreported these behaviors, either intentionally or unintentionally.

Prior to the test, patients were instructed to undergo a 6-min walking test. The walking distance was measured in meters. The results were classified as I class—426–550 m, II class—301–425 m, III class—151–300 m, and VI class—≤150 m. On the test day, the patients performed a spirometry, then a warm up for 3 min. The electrocardiographic exercise testing took for 8–12 min and the recovery phase lasted 5 min more. After the CPET completion, the following parameters were measured: VO_2_ max, METs, O_2_ pulse, and heart rate.

### 2.4. Radiofrequency Ablation

RFA is a clinically effective and safe technology promoting hyperthermal tissue heating, accompanied by the application of intracardiac echocardiography (ICE). Intracardiac echocardiography (ICE) is typically performed by inserting a catheter through the right femoral vein and advancing it into the right atrium (RA), right ventricle (RV), or transeptally into the left atrium. By placing the ICE ultrasound probe in the RA, an initial long-axis view of the RA, RV, and tricuspid valve can be obtained [22].

RFA is performed using a thin, flexible catheter that is inserted through a blood vessel and guided to the source of the abnormal rhythm in the heart causing arrhythmia. A radiofrequency impulse is then delivered through this catheter, which destroys the tissue responsible for the irregular rhythm [22].

In most cases, RFA is associated with a positive clinical outcome for the patient, ranging from 60% to 80% for paroxysmal AF, depending on the ablation strategy, and from 50% to 60% for persistent AF [29]. RFA as a first-line treatment is a cost-effective strategy for young patients with AF, and as a second-line therapy, it is potentially cost-effective for treating paroxysmal AF in patients [30].

### 2.5. Cardiac Function Measurements

Maximal oxygen uptake (VO_2_ max) represents the maximum rate at which oxygen can be used during intense exercise. It is a key indicator of cardiovascular fitness. Metabolic Equivalent Tasks (METs) is a unit that estimates the amount of oxygen used by the body during physical activity. One MET is equivalent to the energy expended at rest. Oxygen (O_2_ pulse) often refers to the oxygen consumed (VO_2_ max) or the oxygen content in the blood. Heart Rate (HR) is the number of heart beats per minute. Left Ventricular End-Diastolic Volume (LVEDV) stands for the volume of blood in the left ventricle at the end of filling (diastole). Left Ventricular End-Systolic Volume (LVESV) is the volume of blood remaining in the left ventricle after contraction (systole). Left Ventricular End-Diastolic Size (LVEDS) is the size of the left ventricle at the end of diastole. Left Ventricular End-Systolic Size (LVESS) is the size of the left ventricle at the end of systole. N-terminal pro–B-type natriuretic peptide (Nt-proBNP) is a key biomarker used to assess heart function, particularly in diagnosing and managing heart failure. Elevated levels of Nt-proBNP indicate that the heart is under stress and struggling to pump blood efficiently.

VO_2_ max and METs assess cardiovascular fitness by measuring the maximum oxygen uptake and energy expenditure during physical activity. HR reflects the heart rate, crucial for understanding the heart’s response to exercise. LVEDV and LVESV represent the blood volume in the left ventricle at the end of diastole and systole, respectively, revealing the heart’s pumping efficiency. LVEDS and LVESS measure the size of the left ventricle at these same points, further illustrating the heart’s structural and functional capacity during the cardiac cycle. Nt-proBNP is a biomarker for heart failure, indicating stress on the heart. Together, these metrics provide a comprehensive overview of cardiac health and function.

### 2.6. Statistical Methods

Continuous variables were reported as either mean ± standard deviation or median with interquartile range (IQR). Categorical variables were presented as absolute numbers and percentages. Patients were categorized into two groups based on measurements before the RFA and at the 3-month follow-up. Differences in clinical, morphological, functional, and biochemical measures were compared between groups and within groups (baseline to 3 months) using appropriate statistical tests: two-tailed *t*-test for dependent or independent samples, chi-square test, and linear mixed models.

The normality of the distribution was assessed using the Shapiro–Wilk test. A *p*-value of less than 0.05 was considered statistically significant. All analyses were conducted using IBM SPSS Statistics version 27.

Binominal variables standing for obesity, hypertension, and cholesterol status were determined based on specific clinical criteria. Patients were classified as either having obesity (BMI ≥ 30) or not having obesity (BMI < 30), following the World Health Organization (WHO) guidelines [31]. Hypertension was defined as systolic blood pressure ≥ 130 mmHg or diastolic blood pressure ≥ 80 mmHg, while normal blood pressure was below these values.

## 3. Results

Table 1 presents the baseline characteristics of the study population before the RFA procedure (*N* = 104). The cohort was predominantly male (61%, *p* = 0.031), with an average age of 46 ± 13 years (median 47 years, IQR: 37–55 years, *p* = 0.010). Obesity was prevalent in 74% of participants (*p* < 0.001), while hypertension affected 57% (*p* = 0.170). LP(a) levels average was 18 ± 15 mg/dL (IQR: 13 [8–19 mg/dL]), significantly below the normal threshold of 22 mg/dL (*p* < 0.001).

Cardiac function measures revealed significant deviations from normal. The average LVEDV was 116 ± 36 (range: 62–150), indicating substantial cardiac stress (*p* < 0.001). LVESV average was 58 ± 26 (range: 21–61, *p* < 0.001), and the LVEDS was 4.9 ± 1.1 cm (range: 4.2–5.8 cm, *p* < 0.001). LVESS was 3.7 ± 1.2 cm (range: 2.5–4 cm, *p* < 0.001). Indexed Volume of the Left Atrium (IVLA) average was 30 ± 7 (range: 16–34 cm^3^, *p* < 0.001). The Left Ventricular Lateral Wall Velocity (S’lat) average was 6.6 cm/s (IQR: 4.3–6.6 cm/s), below the normal range (7.5–12.3 cm/s, *p* < 0.001). The Medial Left Ventricular Wall Velocity (S’med) average was 6.2 cm/s (IQR: 4.0–6.2 cm/s), with no statistically significant difference (*p* = 0.170).

The findings in Table 2 show a significant increase in VO_2_ max and METs across all post-RFA demographics. Despite the youth parameters, highly active age groups (30–39 years) demonstrated around 60% improvement in VO_2_ max and METs, indicating an increase in exercise tolerance post-procedure. Middle-aged and older groups displayed similar trend, with both males and females showing elevated METs after RFA. The older age-group was linked to a more moderate increase in the indicators.

The linear mixed models before and after RFA analysis showed significant improvements across multiple measures (Table 3). VO_2_ max showed enhanced aerobic capacity, indicating a significant increase from 11.5 ± 4.4 mL/kg/min to 18.0 ± 4.5 mL/kg/min (*p* < 0.001). O_2_ pulse also improved from 7.2 ± 2.6 mL/beat to 11.0 ± 3.4 mL/kg/min (*p* < 0.001). The distance covered in the 6-min walking test showed improved exercise endurance, from 306 ± 82 m to 400 ± 48 m (*p* < 0.001). METs increased from 4.4 ± 1.6 to 8.0 ± 1.3 (*p* < 0.001), indicating a positive cardiac adaptation. Furthermore, the heart rate at peak exercise decreased from 175.5 ± 18.6 to 147.2 ± 12.3 beats per minute (*p* < 0.001), indicating improved cardiovascular response. There was also a significant reduction in Nt-proBNP levels, from 1357 ± 1182 to 415 ± 339 pg/mL (*p* < 0.001), suggesting decreased cardiac stress and improved heart function. Interestingly, eighty percent of patients returned to sinus rhythm post-ablation, which likely contributed to the substantial improvements in exercise tolerance.

**Figure 1:**
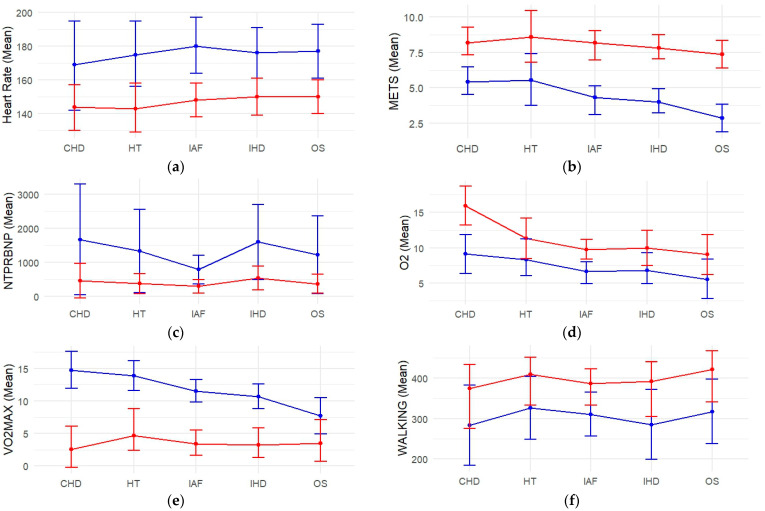
The changes in various CPET and other cardiac indicators means and CIs before (blue line) and after (red line) ablation: (**a**) Heart rate; (**b**) METs; (**c**) Nt-proBNP; (**d**) O_2_ pulse; (**e**) Vo2 max; (**f**) 6-min walking test.

Figure 1 depicts the changes in various CPET and other cardiac indicators before and after ablation, stratified by different comorbidities. The values are given as means (scaled to the right) with standard deviations (SD, scaled to the left). The results are statistically significant, with *p*-values < 0.001 for all variables.

Across all comorbidities, there was a significant reduction in heart rate post-ablation, as shown in Figure 1a. This indicated an overall improvement in cardiac function and better rhythm control, notable in patients with hypertension (HT)—18%, ischemic heart disease (IHD)—15%, coronary heart disease (CHD)—15%, idiopatic atrial fibrillation (IAF)—18% (the highest initial heart rate), and open surgery (OS)—15%. An elevated HR before the procedure reflected the typical tachycardia associated with AF. The drop in the cohort’s HR could indicate successful rhythm control or even a return to normal sinus rhythm in certain cases.

The pre-procedure METs values show that many patients had a reduced ability to perform physical activities, likely due to the limitations imposed by AF. Significant improvements in METs post-ablation indicated better exercise capacity associated with metabolic efficiency (Figure 1b). Patients with open surgery had the lowest baseline METs, showing the most considerable relative improvement (61%). Patients with HT demonstrated an increase of 35%, IHD—48%, CHD—47%, IAF—47%. The increase in means suggested a significant improvement in exercise capacity, indicating that the procedure was generally successful in enhancing patients’ functional status. The reduction in SD implies that this improvement was fairly uniform across the patient group.

The Figure 1c demonstrated a dramatic around 3-fold reduction in Nt-proBNP levels across all post-ablation conditions, reflecting decreased cardiac stress and improved heart function. Interestingly, the wide range of pre-procedure from 200 to 5812 and SD = 1182 indicate significant variability in Nt-proBNP levels, suggesting varying degrees of heart failure or cardiac stress among the heterogeneous population. Patients with the higher Nt-proBNP values were likely to experience advanced heart failure or more significant cardiac stress, aligning with worse clinical outcomes or severe symptoms. On the other hand, post-procedure values decreased from 65 to 2108, SD = 339, indicated overall reduction in cardiac stress or heart failure severity after RFA.

Before the procedure, O_2_ pulse values showed a reduced capacity for oxygen uptake, which was consistent with patients experiencing AF, leading to impaired exercise tolerance and overall reduced cardiorespiratory fitness and oxygen utilization, likely due to improved cardiac function and overall cardiovascular efficiency following the intervention. Oxygen consumption shown in the Figure 1d, significantly advanced in all groups. The most substantial progress was seen in patients with CHD—43%. The rest are HT—26%, IHD—32%, IAF—32%, OS—39%.

The data indicated that all groups experienced an increase in VO_2_ max after the intervention, with the most significant improvement observed in patients with OS at 98.59%, followed by CHD at 64.88%, shown in Figure 1e, as well as IAF—48%, IHD—50%, HT—37%. A higher VO_2_ max typically correlates with better exercise tolerance. Post-procedure, patients were able to engage in physical activities with less fatigue and breathlessness.

Significant improvements in the walking test across indicated enhanced functional capacity (Figure 1f). Patients with open surgery showed the most substantial absolute increase in walking distance, from 318 m to 421 m, while HT patients presented 25% distance improvement, IHD—37%, CHD—32%, and IAF—25%. This improvement is a positive indicator that the patient’s cardiovascular system can now handle more physical stress without adverse symptoms. The ability to walk longer distances without significant discomfort or fatigue is directly linked to an improved QoL. Patients may find it easier to engage in daily activities, which can also positively impact their mental and emotional well-being.

## 4. Discussion

The study prospectively observed improvements of CPET indicators from baseline to 3-months post RFA changes in VO_2_ max, O_2_, 6-min walking test, METs, heart rate, Nt-proBNP, and established predictors of heart failure. The before-and-after changes of these indicators were analyzed by linear mixed model, providing robust and versatile insights into both average effects and individual responses to the treatment. Following RFA in patients with AF, significant improvements in cardiac function and hemodynamics were observed. Specifically, there was a reduction in mitral and tricuspid regurgitation, leading to enhanced valvular function. The size of the left atrium, including the end-diastolic and end-systolic volumes, decreased and were accompanied by a reduction in atrial natriuretic peptide levels, thus indicating reduced volume overload and improved atrial function. Additionally, pulmonary artery systolic pressure decreased, suggesting a reduction in pulmonary hypertension. Furthermore, improvements were seen in myocardial strain parameters, including increased lateral and medial strain velocities (S’lat and S’med), reflecting enhanced myocardial function and overall cardiac performance.

Despite initial concerns, the patients achieved improvements in VO_2_ max, O_2_, 6-min walking test, METs, heart rate, Nt-proBNP, and overall QoL 3 months following ablation. The most substantial functional benefits were observed in younger male patients, who maintained SR, had preserved early left atrial appendage activation, and showed a marked increase in the latter’s outflow velocity compared to their baseline measurements, playing a critical role in predicting post-ablation success and functional recovery.

Several recent RCTs evaluating the impact of structured exercise and rehabilitation programs on patients with AF, both before and after ablation, provided insights into how physical activity influences cardiopulmonary parameters. The ACTIVE-AF trial evaluated the impact of a supervised and at-home exercise program aimed at reducing AF symptom severity and AF recurrence, supporting prescription of physical exercises that target cardiorespiratory functioning as a primary intervention against modifiable risk factors for AF patients [8]. Another OPPORTUNITY trial evaluating the impact of a 12-week high-intensity interval training intervention demonstrated positive effects on functional capacity and disease-specific QoL among AF patients [29].

The 2023 editorial update of the randomized trial showed that a 150-min-per-week exercise program doubled the likelihood of patients being AF-free after 12 months, with a notable improvement in symptoms. The authors argued that sufficient evidence exists to incorporate exercise programs into routine clinical practice. They advocated for broader implementation and innovation in delivery methods, despite current limitations in monitoring and program accessibility. The editorial called for clinicians to proactively recommend exercise to AF patients and to explore or establish relevant programs, supporting these initiatives with real-world evidence [30].

It is known that RFA effectively treats AF, but recurrences remain a significant concern. An 18-year long term study revealed about a 2% return rate for all AF types during the 2-year post-procedure [31]. Factors influencing recurrence included AF type, procedural success, patient age, and underlying cardiac conditions. Meta-analyses and long-term studies indicate that while RFA reduces AF burden, the recurrence rate varies depending on these factors, highlighting the need for ongoing refinement in ablation techniques and patient management [31,32,33,34,35].

Numerous RCTs, like MANTRA-PAF, CASTLE-AF, RAAFT-2, and various prospective studies like AFEQT-OS, have revealed a common tendency of significant QoL post-RFA improvements in patients with AF. These studies have consistently shown a reduction in symptoms, enhanced physical activity, decreased emotional stress, and overall better health outcomes. For example, the CASTLE-AF trial demonstrated particularly notable improvements in quality of life among patients with coexisting heart failure. The MANTRA-PAF confirmed that the long-term outcomes of RFA significantly surpass those of medical therapy in terms of quality-of-life enhancement. Other studies reinforce these findings, highlighting sustained improvements post-RFA, particularly in patients with recurrent, drug-refractory AF [31,35,36,37,38,39,40].

### Limitations

It can be argued that studying cardiopulmonary exercise parameters in patients with atrial fibrillation before and after radiofrequency ablation is a relatively underexplored topic. While radiofrequency ablation is commonly used to treat atrial fibrillation, and there are numerous studies evaluating its effectiveness, research focusing specifically on cardiopulmonary exercise parameters in the context of this procedure is less developed. Investigating how ablation impacts patients’ physical activity, how they adapt to exercise post-procedure, and the long-term effects on their cardiopulmonary status, as well as differences among various patient subgroups, could provide valuable insights into how effective treatment of atrial fibrillation through ablation influences physical activity and cardiopulmonary health. All patients were interviewed regarding their smoking habits and medication use before testing, but we acknowledge the possibility that some may not have fully disclosed this information.

As a single-centered study, replication at other cities will be beneficial to confirm these findings. Approximately one-fifth of the patients underwent the follow-up measurements. Long-distance residence could be a potential confounder. Patients who live far away from the treatment center might be less likely to return for follow-up CPET due to the inconvenience or cost of travel. Generalizability might also take place among the significant number of patients who had complications or no improvement as a primary reason to skip follow-up. Strategies to increase the participation interest in exercise-based research may be needed to increase the number of facilities in the regions to perform 3-, 6-, and 12-months follow-ups. Authors should explore the implications of their results in relation to the existing literature and the hypotheses they formulated. It is essential to contextualize the findings within the broader scope of the field. Additionally, suggestions for future research avenues should be addressed.

## 5. Conclusions

This study of a cohort of patients with persistent atrial fibrillation undergoing radiofrequency ablation resulted in functional improvements, namely in CPET indicators such as VO_2_ max, METs, O_2_ pulse, heart rate, and 6-min walking test. As well, improvements were reported in Nt-proBNP analysis. These findings were achieved with minimal risks of medical complications. This study highlights importance of longitudinal follow-up in patients with AF and HF.

## Figures and Tables

**Table 1:** Baseline characteristics.

Variable	Abs, (%) *N* = 104 Mean ± sd/Median, Q1–Q3	*p*-Value
Males	63 (61%)	0.031
Age (years)	46 ± 13	0.010
Obesity > 30 BMI	77 (74%)	<0.001
Hypertension	59 (57%)	0.170
LVEF	39 (38%)	<0.001
LP(a) < 22	18 ± 15	<0.001
LVEDV (62–150)	83 (80%) 116 ± 36	<0.001
LVESV (21–61)	69 (66%) 58 ± 26	<0.001
LVEDS (4.2–5.8)	31 (30%) 4.9 ± 1.1	<0.001
LVESS (2.5–4)	53 (51%) 3.7 ± 1.2	<0.001
IVLA (16–34)	84 (81%) 30 ± 7	<0.001
S’lat (7.5–12.3)	34 (33%) 6.6 [4.3–6.6]	<0.001
S’med (6.6–10)	45 (43%) 6.2 [4.0–6.2]	0.170

Abbreviations: LVEF—left ventricular ejection fraction; Lp(a)—Lipoprotein (a); LVEDV—Left Ventricular End-Diastolic Volume; LVESV—Left Ventricular End-Systolic Volume; LVEDS—Left Ventricular End-Diastolic Size; LVESS—Left Ventricular End-Systolic Size; IVLA—Indexed Volume of the Left Atrium; S’lat—Left Ventricular Lateral Wall Velocity; S’med—Medial Left Ventricular Wall Velocity.

**Table 2:** Age and gender stratification of CPET indicators.

Age Categories	Before		After	
	Male	Female	Male	Female
18–29	16 ± 1.2 VO_2_ max	14 ± 2.9 VO_2_ max	27 ± 3.9 VO_2_ max	23 ± 2.7 VO_2_ max
	5.2 METs	5.5 METs	8.1 METs	8.3 METs
30–39	12 ± 4.3 VO_2_ max	11 ± 5.0 VO_2_ max	20 ± 3.5 VO_2_ max	18 ± 2.6 VO_2_ max
	5.4 METs	4.2 METs	8.9 METs	8.0 METs
40–49	12 ± 4.3 VO_2_ max	11 ± 5.8 VO_2_ max	18 ± 2.9 VO_2_ max	16 ± 4.4 VO_2_ max
	4.6 METs	4.2 METs	8.4 METs	8.3 METs
50–59	12 ± 3.9 VO_2_ max	10 ± 3.3 VO_2_ max	17 ± 2.5 VO_2_ max	15 ± 2,7 VO_2_ max
	4.2 METs	3.6 METs	7.9 METs	7.2 METs
60+	8 ± 3.7 VO_2_ max	8 ± 2.7 VO_2_ max	13 ± 2.5 VO_2_ max	13 ± 3.9 VO_2_ max
	3.3 METs	3.5 METs	7.4 METs	7.1 METs

Abbreviations: VO_2_ max—Maximal oxygen consumption; METs—Metabolic equivalent of task.

**Table 3:** Linear mixed models evaluation of change in indicators before and after ablation.

Variable	Observation Stage		*p*-Value
	Before	After	
VO_2_ max	11.5 ± 4.4	18.0 ± 4.5	<0.001
O_2_ pulse	7.2 ± 2.6	11.0 ± 3.4	<0.001
Walking test	306 ± 82	400 ± 48	<0.001
METs	4.4 ± 1.6	8.0 ± 1.3	<0.001
Heartrate	175.5 ± 18.6	147.2 ± 12.3	<0.001
Nt-proBNP	1357 ± 1182	415 ± 339	<0.001

Abbreviations: VO_2_ max—Maximal oxygen consumption; METs—Metabolic equivalent of task; O_2_ pulse—oxygen pulse; Walking test—6-min walking test; NtProBNP—N-terminal pro–B-type natriuretic peptide.

## Data Availability

Data supporting the reported results are not publicly available due to privacy and ethical restrictions but can be made available by the corresponding author upon reasonable request.
